# One-Dimensional Organic-Inorganic Nanocomposite Synthesized with Single-Walled Carbon Nanotube Templates

**DOI:** 10.3390/ma7085858

**Published:** 2014-08-13

**Authors:** Wei Li, Aili Wei, Huaiping Zhang, Dojin Kim

**Affiliations:** 1 Department of Material Science and Engineering, Taiyuan University of Technology, Taiyuan 030024, China; E-Mails: weiailidd@126.com (A.W.); huaipingzhang@gmail.com (H.Z.); 2 Department of Material Science and Engineering, Chungnam National University, Deajeon 305764, Korea

**Keywords:** nanocomposite, carbon nanotube, poly(acrylic acid)

## Abstract

This study reports on single-walled carbon nanotubes (SWCNT) as templates for the preparation of 1D porous organic-inorganic hybrid composites. The *in situ* deposited SWCNT were sputter coated with Sn metal and thermally oxidized in air to form a SnO_2_/SWCNT nanowire framework on SiO_2_/Si substrate. Poly(acrylic acid) (PAA) was coated onto this scaffold through UV light-induced radical polymerization, which resulted in the final formation of hybrid composites. The structures of hybrid composites were investigated by scanning electron microscopy, transmission electron microscopy, infrared spectroscopy, and Raman spectroscopy. The results show that PAA was successfully coated and the structural advantage of nanowire was fairly maintained, which indicates that this framework is very stable for organic functionalization in solution. The simplicity of this method for the formation of porous organic-inorganic hybrid composites provides a potential application for nanoelectronic devices.

## 1. Introduction

One-dimensional (1D) nanostructured materials have elicited significant attention because of their unique mechanical, optical, and electrical properties [1]. Template synthesis is one of the most frequent routes to synthesize 1D nanostructure. This route usually involves the use of a structure-directing reagent that facilitates the materials to adopt the desired structures [2], and thus, the premise of successful fabrication is selecting suitable template materials. Carbon nanotubes (CNTs) have been recognized as unique templates because of their structural advantages, such as their small size ranging in diameter from less than one nanometer to dozens of nanometers and extremely high length-to-diameter ratio ranging from 30 to several thousand [3]. Various 1D nanomaterials are prepared using CNT templates, but most synthesis methods are based on solution chemical methods [3,4,5,6,7]. These chemical methods usually suffer from poor nanostructure quality. Specifically, when transferred from solutions to substrates, these 1D nanocomposites easily aggregate due to the capillary force or van der Waals attraction, resulting in the loss of structural advantage. The situation might be changed if CNT templates have already formed a stable scaffold on substrate before mixing with solutions. According to this hypothesis, we designed a novel method to fabricate organic-inorganic nanocomposites based on CNT template.

**Figure 1:**
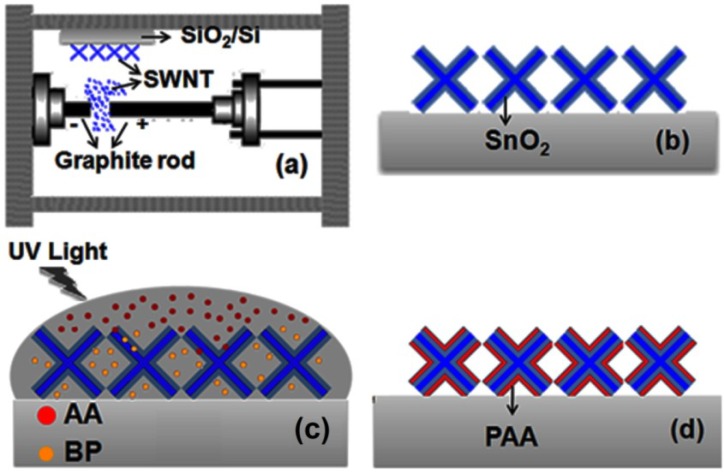
Schematic diagrams of the fabrication procedure for Poly(acrylic acid) (PAA)/SnO_2_/single-walled carbon nanotubes (SWCNT) nanocomposites. (**a**) *In* *situ* deposition of SWCNT; (**b**) structural diagram of SnO_2_/SWCNT; (**c**) diagram of UV light-induced radical polymerization of acrylic acid (AA) using benzophenone (BP) initiator; and (**d**) structural diagram of PAA/SnO_2_/SWCNT nanocomposites.

The fabrication procedures for nanocomposites are schematically summarized in Figure 1. The synthesized single walled carbon nanotubes (SWCNTs) directly flowed onto the substrate to form a SWCNT film when the arc was discharged (Figure 1a). A thin tin metal layer was deposited over the SWCNT film by sputtering and thermally oxidized in air to form composite nanowires (Figure 1b). Finally, this SnO_2_/SWCNT composite was further developed to form 1D organic-inorganic nanocomposite by coating a thin layer of poly(acrylic acid) (PAA) through UV light-induced radical polymerization (Figure 1c,d).

## 2. Results and Discussion

**Figure 2:**
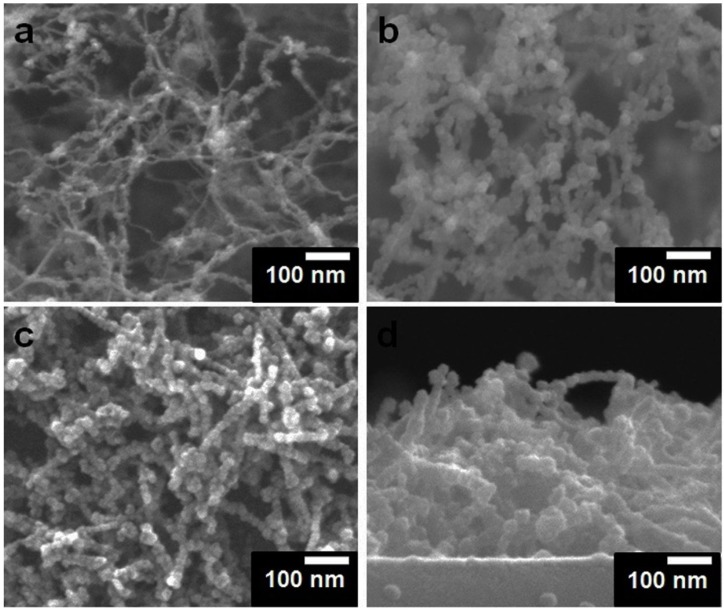
SEM pictures of (**a**) SWCNT; (**b**) SnO_2_/SWCNT; (**c**) PAA/SnO_2_/SWCNT nanocomposites; and (**d**) the cross-section view of PAA/SnO_2_/SWCNT nanocomposites.

Figure 2 shows the SEM images of SWCNT template and PAA/SnO_2_/SWCNT nanocomposite film. The SWCNTs were entangled with each other and exhibited highly porous structures (Figure 2a). The porosity of these *in situ* deposited SWCNT was estimated to be up to 95% and mainly caused by the steric hindrance effect created when the SWCNT flowed to the substrate and seat layer by layer [8]. The morphology of SnO_2_/SWCNT is shown in Figure 2b. The shape of the composite appeared like a rope of beads instead of a regular tube-like structure, which is closely associated to the poor wetting property of SnO_2_ on SWCNT [8]. Both SnO_2_/SWCNT and PAA/SnO_2_/SWCNT nanocomposites seem to duplicate structure of SWCNT template (Figure 2b,c). The SnO_2_ deposited over SWCNT protected the morphology of SWCNT templates and ensured their application during wet chemical treatment. Notably, the extremely light SWCNT can be easily removed from the substrate. In addition, the hydrophobic nature of SWCNT can contribute to the easy collapse of their spatial structures in polar solution, such as water and methanol [9]. By contrast, PAA grafting did not significantly change the SnO_2_/SWCNT morphology because a drop of solution contained a small amount of AA monomer. Thus, the PAAs were not sufficient to fill the pores or enclose the nanowires. The thickness of PAA/SnO2/SWCNT nanocomposite film was approximately 200 nm to 300 nm (Figure 2d).

**Figure 3:**
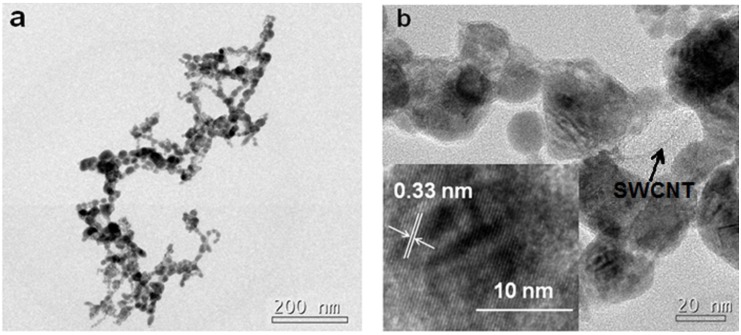
TEM images (**a**) and its high magnification (**b**) of PAA/SnO_2_/SWCNT nanocomposites. Inset of (**b**) shows the (110) planes of SnO_2_.

Figure 3 shows the TEM images of PAA/SnO_2_/SWCNT nanocomposite. The SnO_2_ beads almost completely wrapped the nanotubes, and both SWCNTs and PAA could not be clearly distinguished under low magnification (Figure 3a). However, in a higher-resolution image (Figure 3b), PAA and a few pristine SWCNTs could be observed. The thickness of PAA coated on SnO_2_ was from several nanometers up to 10 nm. This small thickness could not affect the integral structures, which suggests that the morphologies in SEM images did not significantly change. The inter-planar space of 0.33 nm corresponded to the (110) plane of SnO_2_ [10].

**Figure 4:**
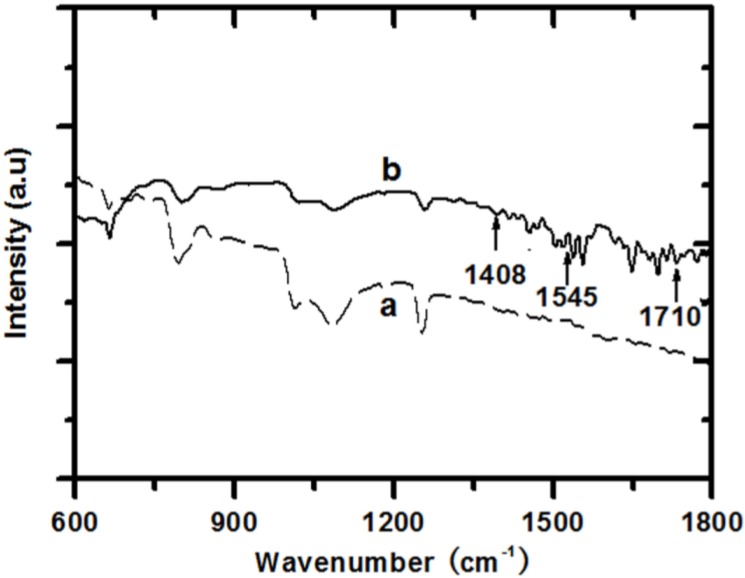
FTIR spectra of (**a**) SnO_2_/SWCNT and (**b**) PAA/SnO_2_/SWCNT nanocomposites.

FTIR characterization is an informative approach to study the functional groups attached on SnO_2_/SWCNTs. Figure 4 shows the FTIR spectra of nanocomposites. After coating, a group of serrate-like absorbance bands clearly appeared in the region of 1400 cm^−1^ to 1800 cm^−1^. The band at 1710 cm^−1^ was attributed to the stretching mode of carbonyl groups, which indicates the presence of PAA on the surface of SnO_2_/SWCNT [11]. The bands at approximately 1400 cm^−1^ to 1560 cm^−1^ were attributed to the stretching mode of C–O and the deformation vibration of hydroxyl. Specifically, the bands at 1408 and 1545 cm^−1^ were due to the binding of the carboxylic acid groups to the surface of SnO_2_ nanobeads to form carboxylate groups [12].

**Figure 5:**
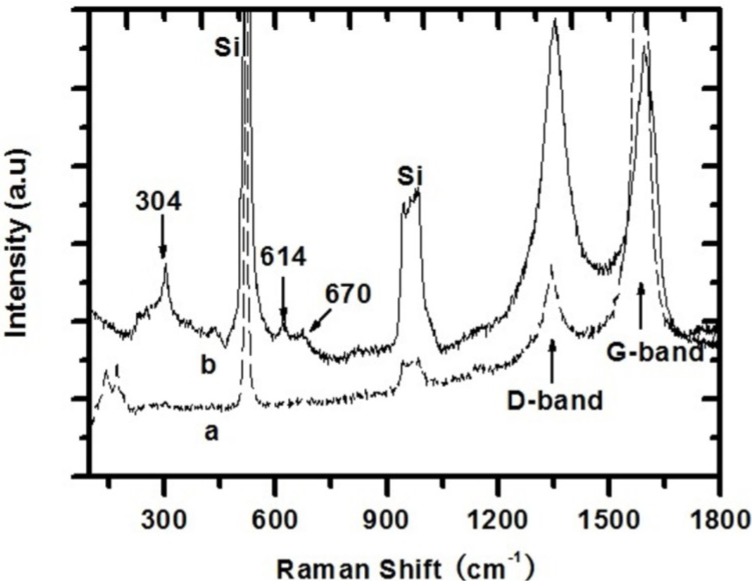
Raman spectra of (**a**) SWCNT and (**b**) PAA/SnO_2_/SWCNT nanocomposites.

Raman spectra as complementary investigation to FTIR are shown in Figure 5. Two distinct peaks associated to the vibration modes of SWCNTs within the region of 1200 cm^−1^ to 1800 cm^−1^ were observed [13]. The peak at 1590 cm^−1^, the so-called G band, corresponded to the sp_2_ vibration of a 2D hexagonal lattice in the graphite; another peak at 1350 cm^−1^, the so-called D band, was associated to glassy carbon or disordered graphite. The intensity of D band significantly increased and the corresponding G band intensity decreased in the spectrum of nanocomposite. In general, the intensity ratio of D to G band is a sign of defect/amorphous carbon concentration in SWCNTs [14]. The D/G intensity ratio of 1.03 for nanocomposite was much higher than that of 0.18 for SWCNTs, which indicates that PAA/SnO_2_ coating created more defects on the SWCNT surface. In addition, the radial breathing mode (RBM) in the range of 100–300 cm^−1^ was present in spectrum of SWCNT, and the tube diameters were approximately 1.4 nm from the RBM frequency [8,15,16]. Furthermore, no RBM signal was present in the spectrum of nano-composite. The possible reason is that PAA/SnO_2_ coating increases the thickness and surface defect of SWCNT bundles, resulting in a reducing of signal [17]. Three bands of SnO_2_ at 304, 614, and 670 cm^−1^ were observed in the nanocomposite spectrum. The band at 304 cm^−1^ corresponded to the infrared-active E_u_ (TO) mode [18,19], and the band at 614 cm^−1^ was associated with the Raman-active A_1g_ mode [18]. The band at 670 cm^−1^ possibly indicated other tin oxide stoichiometries [20].

Raman mode bands at approximately 520 and 950 cm^−1^ in both spectra were associated with the vibration mode of the substrate. Their appearances were due to the high film porosity, which allowed the laser beam to penetrate down to the substrate. Meanwhile, their intensities in the nanocomposite were much higher than those in SWCNTs. This phenomenon was possibly associated with the film thickness because PAA and SnO_2_ coating will reduce the overall film thickness and allow the laser beam to penetrate more easily. Furthermore, no characteristic peaks of PAA were observed [21,22,23]; they probably overlapped with the strong substrate peaks.

## 3. Experimental Section

### 3.1. Synthesis of SnO_2_/SWCNT

The SiO_2_/Si substrates were mounted on the wall of an arc-discharging chamber. When the arc was discharged, the synthesized SWCNT directly flowed onto the substrate and formed a film. The source material for arc discharge was a graphite rod where catalytic metal wires of Ni, Fe, and Mo were inserted. The synthesis was carried out in an optimized condition with an arc current density of 80 A/cm^2^ in H_2_ atmosphere at 400 Torr for 1 min [13]. The SWCNT film was first installed in a sputtering chamber equipped with a tin target. The tin was deposited in Ar gas atmosphere at room temperature and heated in a tube furnace in air (450 °C) for 2 h to form SnO_2_/SWCNT composite nanowires.

### 3.2. Synthesis of PAA/SnO_2_/SWCNT Composite

AA monomer and benzophenone (BP) initiator were purchased from Sigma-Aldrich (Shanghai, China) and used without further purification. The SiO_2_/Si substrate covered by SnO_2_/SWCNT composite nanowires was immersed in BP methanol solution for 20 min and dried for 2 h at room temperature to deposit the BP molecules onto the nanowire surface. A drop of AA solution (5 wt%) was placed on the substrate, and then the substrate was enclosed in a photo-reactor and irradiated by UV light at room temperature. Finally, the nanocomposite film was washed with distilled water and methanol to eliminate the residual monomer, BP initiator, and homopolymer.

### 3.3. Characterizations

Scanning electron microscopy (SEM) measurements were obtained using a JEOL-7000 instrument (Tokyo, Japan) at an accelerating voltage of 15 kV. Transmission electron microscopy (TEM) measurements were carried out using a FEI Tecnai F30 instrument (Eindhoven, The Netherlands) at an accelerating voltage of up to 200 kV. The samples were placed on 300 mesh carbon-coated copper grid. Fourier transform infrared (FTIR) spectroscopy was recorded using a Bio-Rad FTS-175C instrument (Hercules, CA, USA). Raman spectroscopy measurements were recorded in backscattering geometry with a Horiba LabRam HR instrument fitted with a liquid nitrogen-cooled CCD detector (Paris, France). The spectra were collected under ambient conditions using the 514.5 nm line of an argon-ion laser.

## 4. Conclusions

In summary, the PAA was successfully coated onto the surface of SnO_2_/SWCNT nanowires and formed 1D PAA/SnO_2_/SWCNT nanocomposites through UV light-induced radical polymerization. The nanocomposite not only had a chemical affinity property of PAA but also essentially maintained the structural property of SWCNT templates, confirming that the method used in this work is very suitable to prepare a stable 1D template for organic functionalization in solution. In further studies, we will not only focus on the application of PAA/SnO_2_/SWCNT nanocomposite to nanoelectrical devices, such as gas sensors and glucose biosensors, but also utilize this method to make other hybrid nanocomposites, such as polyaniline/SnO_2_/SWCNT.
